# TopHat-Fusion: an algorithm for discovery of novel fusion transcripts

**DOI:** 10.1186/gb-2011-12-8-r72

**Published:** 2011-08-11

**Authors:** Daehwan Kim, Steven L Salzberg

**Affiliations:** 1 Center for Bioinformatics and Computational Biology, 3115 Biomolecular Sciences Building #296, University of Maryland, College Park, MD 20742, USA; 2 McKusick-Nathans Institute of Genetic Medicine, Johns Hopkins University School of Medicine, Broadway Research Building, 733 N Broadway, Baltimore, MD 21205, USA; 3 Department of Medicine, Johns Hopkins University School of Medicine, Baltimore, MD 21205, USA

## Abstract

TopHat-Fusion is an algorithm designed to discover transcripts representing fusion gene products, which result from the breakage and re-joining of two different chromosomes, or from rearrangements within a chromosome. TopHat-Fusion is an enhanced version of TopHat, an efficient program that aligns RNA-seq reads without relying on existing annotation. Because it is independent of gene annotation, TopHat-Fusion can discover fusion products deriving from known genes, unknown genes and unannotated splice variants of known genes. Using RNA-seq data from breast and prostate cancer cell lines, we detected both previously reported and novel fusions with solid supporting evidence. TopHat-Fusion is available at http://tophat-fusion.sourceforge.net/.

## Background

Direct sequencing of messenger RNA transcripts using the RNA-seq protocol [1-3] is rapidly becoming the method of choice for detecting and quantifying all the genes being expressed in a cell [4]. One advantage of RNA-seq is that, unlike microarray expression techniques, it does not rely on pre-existing knowledge of gene content, and therefore it can detect entirely novel genes and novel splice variants of existing genes. In order to detect novel genes, however, the software used to analyze RNA-seq experiments must be able to align the transcript sequences anywhere on the genome, without relying on existing annotation. TopHat [5] was one of the first spliced alignment programs able to perform such *ab initio *spliced alignment, and in combination with the Cufflinks program [6], it is part of a software analysis suite that can detect and quantify the complete set of genes captured by an RNA-seq experiment.

In addition to detection of novel genes, RNA-seq has the potential to discover genes created by complex chromosomal rearrangements. 'Fusion' genes formed by the breakage and re-joining of two different chromosomes have repeatedly been implicated in the development of cancer, notably the *BCR*/*ABL1 *gene fusion in chronic myeloid leukemia [7-9]. Fusion genes can also be created by the breakage and rearrangement of a single chromosome, bringing together transcribed sequences that are normally separate. As of early 2011, the Mitelman database [10] documented nearly 60,000 cases of chromosome aberrations and gene fusions in cancer. Discovering these fusions via RNA-seq has a distinct advantage over whole-genome sequencing, due to the fact that in the highly rearranged genomes of some tumor samples, many rearrangements might be present although only a fraction might alter transcription. RNA-seq identifies only those chromosomal fusion events that produce transcripts. It has the further advantage that it allows one to detect multiple alternative splice variants that might be produced by a fusion event. However, if a fusion involves only a non-transcribed promoter element, RNA-seq will not detect it.

In order to detect such fusion events, special purpose software is needed for aligning the relatively short reads from next-generation sequencers. Here we describe a new method, TopHat-Fusion, designed to capture these events. We demonstrate its effectiveness on six different cancer cell lines, in each of which it found multiple gene fusion events, including both known and novel fusions. Although other algorithms for detecting gene fusions have been described recently [11,12], these methods use unspliced alignment software (for example, Bowtie [13] and ELAND [14]) and rely on finding paired reads that map to either side of a fusion boundary. They also rely on known annotation, searching known exons for possible fusion boundaries. In contrast, TopHat-Fusion directly detects individual reads (as well as paired reads) that span a fusion event, and because it does not rely on annotation, it finds events involving novel splice variants and entirely novel genes.

Other recent computational methods that have been developed to find fusion genes include SplitSeek [15], a spliced aligner that maps the two non-overlapping ends of a read (using 21 to 24 base anchors) independently to locate fusion events. This is similar to TopHat-Fusion, which splits each read into several pieces, but SplitSeek supports only SOLiD reads. A different strategy is used by Trans-ABySS [16], a *de novo *transcript assembler, which first uses ABySS [17] to assemble RNA-seq reads into full-length transcripts. After the assembly step, it then uses BLAT [18] to map the assembled transcripts to detect any that discordantly map across fusion points. This is a very time-consuming process: it took 350 CPU hours to assemble 147 million reads and > 130 hours for the subsequent mapping step. ShortFuse [19] is similar to TopHat in that it first uses Bowtie to map the reads, but like other tools it depends on read pairs that map to discordant positions. FusionSeq [20] uses a different alignment program for its initial alignments, but is similar to TopHat-Fusion in employing a series of sophisticated filters to remove false positives.

We have released the special-purpose algorithms in TopHat-Fusion as a separate package from TopHat, although some code is shared between the packages. TopHat-Fusion is free, open source software that can be downloaded from the TopHat-Fusion website [21].

## Results

We tested TopHat-Fusion on RNA-seq data from two recent studies of fusion genes: (1) four breast cancer cell lines (BT474, SKBR3, KPL4, MCF7) described by Edgren *et al. *[12] and available from the NCBI Sequence Read Archive [SRA:SRP003186]; and (2) the VCaP prostate cancer cell line and the Universal Human Reference (UHR) cell line, both from Maher *et al. *[11]. The data sets contained > 240 million reads, including both paired-end and single-end reads (Table 1). We mapped all reads to the human genome (UCSC hg19) with TopHat-Fusion, and we identified the genes involved in each fusion using the RefSeq and Ensembl human annotations.

**Table 1:** RNA-seq data used to test TopHat-Fusion

Data source	Sample ID	Read type	Fragment length	Read length	Number of fragments (or reads)
Edgren *et al. *[12]	BT474	Paired	100, 200	50	21,423,697
Edgren *et al. *[12]	SKBR3	Paired	100, 200	50	18,140,246
Edgren *et al. *[12]	KPL4	Paired	100	50	6,796,443
Edgren *et al. *[12]	MCF7	Paired	100	50	8,409,785
Maher *et al. *[11]	VCaP	Paired	300	50	16,894,522
Maher *et al. *[11]	UHR	Paired	300	50	25,294,164
Maher *et al. *[11]	UHR	Single		100	56,129,471

The data came from two studies, and included four samples from breast cancer cells (BT474, SKBR3, KPL4, MCF7), one prostate cancer cell line (VCaP), and two samples from the Universal Human Reference (UHR) cell line. For paired-end data, two reads were generated from each fragment; thus, the total number of reads is twice the number of fragments.

One of the biggest computational challenges in finding fusion gene products is the huge number of false positives that result from a straightforward alignment procedure. This is caused by the numerous repetitive sequences in the genome, which allow many reads to align to multiple locations on the genome. To address this problem, we developed strict filtering routines to eliminate the vast majority of spurious alignments (see Materials and methods). These filters allowed us to reduce the number of fusions reported by the algorithm from > 100,000 to just a few dozen, all of which had strong support from multiple reads.

Overall, TopHat-Fusion found 76 fusion genes in the four breast cancer cell lines (Table 2; Additional file 1) and 19 in the prostate cancer (VCaP) cell line (Table 3; Additional file 2). In the breast cancer data, TopHat-Fusion found 25 out of the 27 previously reported fusions [12]. Of the two fusions TopHat-Fusion missed (DHX35-ITCH, NFS1-PREX1), DHX35-ITCH was included in the initial output, but was filtered out because it was supported by only one singleton read and one mate pair. The remaining 51 fusion genes were not previously reported. In the VCaP data, TopHat-Fusion found 9 of the 11 fusions reported previously [11] plus 10 novel fusions. One of the missing fusions involved two overlapping genes, ZNF577 and ZNF649 on chromosome 19, which appears to be read-through transcription rather than a true gene fusion.

**Table 2:** Seventy-six candidate fusions reported by TopHat-Fusion in four breast cancer cell lines

SAMPLE ID	Fusion genes (left-right)	Chromosomes (left-right)	5' position	3' position	Spanning reads	Spanning pairs
BT474	*TRPC4AP*-*MRPL45*	20-17	33665850	36476499	2	9
BT474	*TOB1*-*SYNRG*	17-17	48943418	35880750	26	47
SKBR3	***TATDN1-GSDMB***	8-17	125551264	38066175	311	555
BT474	*THRA*-*SKAP1*	17-17	38243102	46384689	28	46
MCF7	***BCAS4-BCAS3***	20-17	49411707	59445685	105	284
BT474	***ACACA-STAC2***	17-17	35479452	37374425	57	59
BT474	*STX16*-*RAE1*	20-20	57227142	55929087	6	24
BT474	*MED1*-*ACSF2*	17-17	37595419	48548386	10	12
MCF7	ENSG00000254868-*FOXA1*	14-14	38184710	38061534	2	22
SKBR3	***ANKHD1-PCDH1***	5-5	139825557	141234002	4	15
BT474	***ZMYND8-CEP250***	20-20	45852972	34078459	10	53
BT474	*AHCTF1*-*NAAA*	1-4	247094879	76846963	10	42
SKBR3	***SUMF1-LRRFIP2***	3-3	4418012	37170638	3	12
KPL4	***BSG-NFIX***	19-19	580779	13135832	12	27
BT474	***VAPB-IKZF3***	20-17	56964574	37922743	4	14
BT474	*DLG2*-*HFM1*	11-1	85195025	91853144	2	10
SKBR3	***CSE1L*-ENSG00000236127**	20-20	47688988	47956855	13	31
MCF7	*RSBN1*-*AP4B1*	1-1	114354329	114442495	6	7
BT474	*MED13*-*BCAS3*	17-17	60129899	59469335	3	14
MCF7	***ARFGEF2-SULF2***	20-20	47538545	46365686	17	20
BT474	*HFM1*-ENSG00000225630	1-1	91853144	565937	2	43
KPL4	*MUC20*-ENSG00000249796	3-3	195456606	195352198	13	46
KPL4	*MUC20*-ENSG00000236833	3-3	195456612	197391649	8	15
MCF7	***RPS6KB1-TMEM49***	17-17	57992061	57917126	4	3
SKBR3	***WDR67-ZNF704***	8-8	124096577	81733851	3	3
BT474	***CPNE1-PI3***	20-20	34243123	43804501	2	6
BT474	ENSG00000229344-*RYR2*	1-1	568361	237766339	1	19
BT474	***LAMP1-MCF2L***	13-13	113951808	113718616	2	6
MCF7	*SULF2*-*ZNF217*	20-20	46415146	52210647	11	32
BT474	*WBSCR17*-*FBXL20*	7-17	70958325	37557612	2	8
MCF7	ENSG00000224738-*TMEM49*	17-17	57184949	57915653	5	6
MCF7	*ANKRD30BL*-*RPS23*	2-5	133012791	81574161	2	6
BT474	ENSG00000251948-*SLCO5A1*	19-8	24184149	70602608	2	6
BT474	***GLB1-CMTM7***	3-3	33055545	32483333	2	6
KPL4	*EEF1DP3*-*FRY*	13-13	32520314	32652967	2	4
MCF7	*PAPOLA*-*AK7*	14-14	96968936	96904171	3	3
BT474	*ZNF185*-*GABRA3*	X-X	152114004	151468336	2	3
KPL4	***PPP1R12A-SEPT10***	12-2	80211173	110343414	3	8
BT474	***SKA2-MYO19***	17-17	57232490	34863349	5	12
MCF7	*LRP1B*-*PLXDC1*	2-17	142237963	37265642	2	5
BT474	*NDUFB8*-*TUBD1*	10-17	102289117	57962592	1	49
BT474	ENSG00000225630-*NOTCH2NL*	1-1	565870	145277319	1	18
SKBR3	***CYTH1-EIF3H***	17-8	76778283	117768257	18	37
BT474	*PSMD3*-ENSG00000237973	17-1	38151673	566925	1	12
BT474	***STARD3-DOK5***	17-20	37793479	53259992	2	10
BT474	***DIDO1-TTI1***	20-20	61569147	36634798	1	10
BT474	***RAB22A-MYO9B***	20-19	56886176	17256205	8	20
KPL4	*PCBD2*-ENSG00000240967	5-5	134259840	99382129	1	32
SKBR3	**RARA-PKIA**	17-8	38465535	79510590	1	5
BT474	*MED1*-*STXBP4*	17-17	37607288	53218672	13	11
KPL4	*C1orf151*-ENSG00000224237	1-3	19923605	27256479	1	5
SKBR3	*RNF6*-*FOXO1*	13-13	26795971	41192773	2	13
SKBR3	*BAT1*-ENSG00000254406	6-11	31499072	119692419	2	30
BT474	*KIAA0825*-*PCBD2*	5-5	93904985	134259811	1	19
SKBR3	*PCBD2*-*ANKRD30BL*	5-2	134263179	133012790	1	5
BT474	ENSG00000225630-*MTRNR2L8*	1-11	565457	10530147	1	35
BT474	*PCBD2*-ENSG00000251948	5-19	134260431	24184146	2	6
BT474	*ANKRD30BL*-ENSG00000237973	2-1	133012085	567103	2	8
KPL4	ENSG00000225972-*HSP90AB1*	1-6	564639	44220780	1	7
BT474	*MTIF2*-ENSG00000228826	2-1	55470625	121244943	1	11
BT474	ENSG00000224905-*PCBD2*	21-5	15457432	134263223	2	7
BT474	***RPS6KB1-SNF8***	17-17	57970686	47021335	48	57
BT474	*MTRNR2L8*-*PCBD2*	11-5	10530146	134263156	1	6
BT474	*RPL23*-ENSG00000225630	17-1	37009355	565697	3	19
BT474	*MTRNR2L2*-*PCBD2*	5-5	79946288	134259832	1	5
SKBR3	ENSG00000240409-*PCBD2*	1-5	569005	134260124	2	4
SKBR3	*PCBD2*-ENSG00000239776	5-12	134263289	127650986	2	3
BT474	ENSG00000239776-*MTRNR2L2*	12-5	127650981	79946277	2	3
BT474	*JAK2*-*TCF3*	9-19	5112849	1610500	1	46
KPL4	***NOTCH1-NUP214***	9-9	139438475	134062675	3	5
BT474	*MTRNR2L8*-*TRBV25OR92*	11-9	10530594	33657801	4	4
BT474	*MTRNR2L8*-*AKAP6*	11-14	10530179	32953468	1	5
BT474	ENSG00000230916-*PCBD2*	X-5	125606246	134263219	1	5
MCF7	ENSG00000226505-*MRPL36*	2-5	70329650	1799907	5	20
SKBR3	***CCDC85C-SETD3***	14-14	100002351	99880270	5	6
BT474	*RPL23*-ENSG00000230406	17-2	37009955	222457168	109	5

The 76 candidate fusion genes found by TopHat-Fusion in four breast cancer cell lines (BT474, SKBR3, KPL4, MCF7), with previously reported fusions [12] shown in boldface. The remaining 51 fusion genes are novel. The fusions are sorted by the scoring scheme described in Materials and methods.

**Table 3:** Nineteen candidate fusions reported by TopHat-Fusion in the prostate cell line

Fusion genes (left-right)	Chromosomes (left-right)	5' position	3' position	Spanning reads	Spanning pairs
***ZDHHC7-ABCB9***	16-12	85023908	123444867	13	69
***TMPRSS2-ERG***	21-21	42879875	39817542	7	285
***HJURP-EIF4E2***	2-2	234749254	233421125	3	9
*VWA2*-*PRKCH*	10-14	116008521	61909826	1	10
*RGS3*-*PRKAR1B*	9-7	116299195	699055	3	11
***SPOCK1-TBC1D9B***	5-5	136397966	179305324	9	31
*LRP4*-*FBXL20*	11-17	46911864	37557613	5	9
***INPP4A-HJURP***	2-2	99193605	234746297	6	12
*C16orf70*-*C16orf48*	16-16	67144140	67700168	2	19
*NDUFV2*-ENSG00000188699	18-19	9102729	53727808	1	35
*NEAT1*-ENSG00000229344	11-1	65190281	568419	1	17
**ENSG00000011405-*TEAD1***	11-11	17229396	12883794	7	9
***USP10-ZDHHC7***	16-16	84733713	85024243	1	22
***LMAN2-AP3S1***	5-5	176778452	115202366	15	2
*WDR45L*-ENSG00000224737	17-17	80579516	30439195	1	33
***RC3H2-RGS3***	9-9	125622198	116299072	3	11
*CTNNA1*-ENSG00000249026	5-5	138145895	114727795	1	12
*IMMTP1*-*IMMT*	21-2	46097128	86389185	1	50
ENSG00000214009-*PCNA*	X-20	45918367	5098168	1	24

Nineteen candidate fusions found by TopHat-Fusion in the VCaP prostate cell line, with previously reported fusions [11] indicated in boldface. Fusion genes are sorted according to the scoring scheme described in Materials and methods.

**Figure 1:**
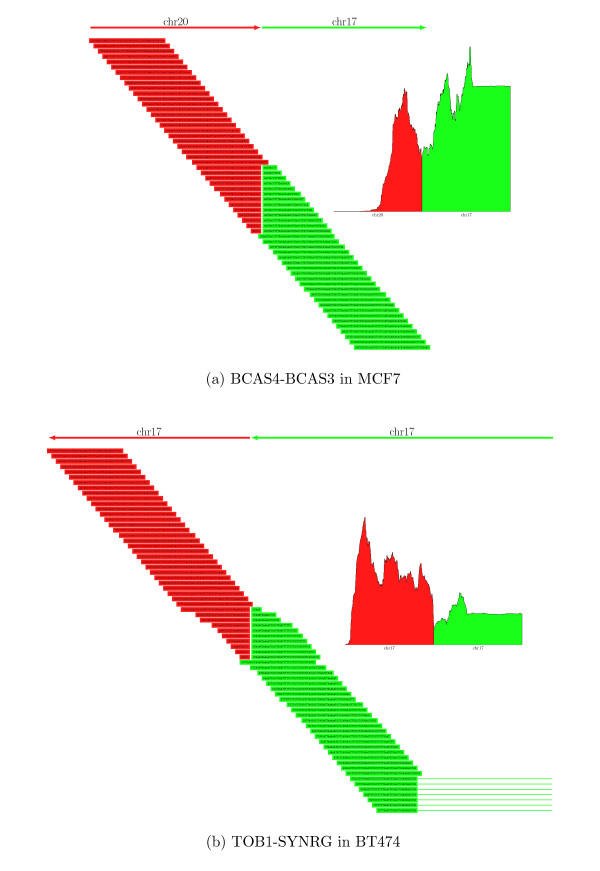
**Read distributions around two fusions: *BCAS4*-*BCAS3 *and *TOB1*-*SYNRG***. **(a) **Sixty reads aligned by TopHat-Fusion that identify a fusion product formed by the *BCAS4 *gene on chromosome 20 and the *BCAS3 *gene on chromosome 17. The data contained more reads than shown; they are collapsed to illustrate how well they are distributed. The inset figures show the coverage depth in 600-bp windows around each fusion. **(b) ***TOB1 *(ENSG00000141232)-*SYNRG *is a novel fusion gene found by TopHat-Fusion, shown here with 70 reads mapping across the fusion point. Note that some of the reads in green span an intron (indicated by thin horizontal lines extending to the right), a feature that can be detected by TopHat's spliced alignment procedure.

Figure 1 illustrates two of the fusion genes identified by TopHat-Fusion. Figure 1a shows the reads spanning a fusion between the *BCAS3 *(breast carcinoma amplified sequence 3) gene on chromosome 17 (17q23) and the *BCAS4 *gene on chromosome 20 (20q13), originally found in the MCF7 cell line in 2002 [22]. As illustrated in the figure, many reads clearly span the boundary of the fusion between chromosomes 20 and 17, illustrating the single-base precision enabled by TopHat-Fusion. Figure 1b shows a novel intra-chromosomal fusion product with similarly strong alignment evidence that TopHat-Fusion found in BT474 cells. This fusion merges two genes that are 13 megabases apart on chromosome 17: *TOB1 *(transducer of ERBB2, ENSG00000141232) at approximately 48.9 Mb; and *SYNRG *(synergin gamma) at approximately 35.9 Mb.

### Single versus paired-end reads

Using four known fusion genes (*GAS6*-*RASA3*, *BCR*-*ABL1*, *ARFGEF2*-*SULF2*, and *BCAS4*-*BCAS3*), we compared TopHat-Fusion's results using single and paired-end reads from the UHR data set (Table 4). All four fusions were detected using either type of input data. Although Maher *et al. *[11] reported much greater sensitivity using paired reads, we found that the ability to detect fusions using single-end reads, when used with TopHat-Fusion, was sometimes nearly as good as with paired reads. For example, the reads aligning to the *BCR*-*ABL1 *fusion provided similar support using either single or paired-end data (Additional file 3). Among the top 20 fusion genes in the UHR data, 3 had more support from single-end reads and 9 had better support from paired-end reads (Additional file 4). Note that longer reads might be more effective for detecting gene fusions from unpaired reads: Zhao *et al. *[23] found 4 inter-chromosomal and 3 intra-chromosomal fusions in a breast cancer cell line (HCC1954), using 510,703 relatively long reads (average 254 bp) sequenced using 454 pyrosequencing technology. Very recently, the FusionMap system [24] was reported to achieve better results, using simulated 75-bp reads, on single-end versus paired-end reads when the inner mate distance is short.

**Table 4:** Comparisons of results from using single-end and paired-end reads for finding fusions

Read type	Fusion genes (left-right)	Chromosomes (left-right)	5' position	3' position	Spanning reads (RPM)	Spanning pairs
Single	*GAS6*-*RASA3*	13-13	114529968	114751268	15 (0.267)	
Paired	*GAS6*-*RASA3*	13-13	114529968	114751268	10 (0.198)	43
Single	*BCR*-*ABL1*	22-9	23632599	133655755	6 (0.107)	
Single	*BCR*-*ABL1*	22-9	23632599	133729450	3 (0.053)	
Paired	*BCR*-*ABL1*	22-9	23632599	133655755	2 (0.040)	7
Paired	*BCR*-*ABL1*	22-9	23632599	133729450	3 (0.059)	10
Single	*ARFGEF2*-*SULF2*	20-20	47538548	46365683	17 (0.302)	
Paired	*ARFGEF2*-*SULF2*	20-20	47538545	46365686	10 (0.198)	30
Single	*BCAS4*-*BCAS3*	20-17	49411707	59445685	25 (0.445)	
Paired	*BCAS4*-*BCAS3*	20-17	49411707	59445685	13 (0.257)	145

Comparisons of single-end and paired-end reads as evidence for gene fusions in the Universal Human Reference (UHR) cell line (a mixture of multiple cancer cell lines), using the known fusions *GAS6*-*RASA3*, *BCR*-*ABL1*, *ARFGEF2*-*SULF2*, and *BCAS4*-*BCAS3*. With TopHat-Fusion's ability to align a read across a fusion, the single-end approach is competitive with the paired-end-based approach. RPM is the number of reads that span a fusion per millon reads sequenced. For instance, the RPM of single-end reads in *GAS6*-*RASA3 *is 0.267, which is slightly better than the RPM for paired-end reads. Single-end reads may show higher RPM values than paired-ends in part because single-end reads are longer (100 bp) than paired-end reads (50 bp) in these data, and therefore they are more likely to span fusions.

### Estimate of the false positive rate

In order to estimate the false positive rate of TopHat-Fusion, we ran it on RNA-seq data from normal human tissue, in which fusion transcripts should be absent. Using paired-end RNA-seq reads from two tissue samples (testes and thyroid) from the Illumina Body Map 2.0 data [ENA: ERP000546] (see [25] for the download web page), the system reported just one and nine fusion transcripts in the two samples, respectively. Considering that each sample comprised approximately 163 million reads, and assuming that all reported fusions are false positives, the false positive rate would be approximately 1 per 32 million reads. Some of the reported fusions may in fact be chimeric sequences due to ligation of cDNA fragments [26], which would make the false positive rate even lower. For this experiment, we required five spanning reads and five supporting mate pairs because the number of reads is much higher than those of our other test samples. When the filtering parameters are changed to one read and two mate pairs, TopHat-Fusion predicts 4 and 43 fusion transcripts in the two samples, respectively (Additional file 5).

Because it is also a standalone fusion detection system, we ran FusionSeq (0.7.0) [20] on one of our data sets to compare its performance to TopHat-Fusion. FusionSeq consists of two main steps: (1) identifying potential fusions based on paired-end mappings; and (2) filtering out fusions with a sophisticated filtration cascade containing more than ten filters. Using the breast cancer cell line MCF7, in which three true fusions (*BCAS4*-*BCAS3*, *ARFGEF2*-*SULF2*, *RPS6KB1*-*TMEM49*) were previously reported, we ran FusionSeq with mappings from Bowtie that included discordantly mapped mate pairs. (Note that FusionSeq was designed to use the commercial ELAND aligner, but we used the open-source Bowtie instead.) To do this, we aligned each end of every mate pair separately, allowing them to be aligned to at most two places, and then combined and converted them to the input format required by FusionSeq.

When we required at least two supporting mate pairs for a fusion (the same requirement as for our TopHat-Fusion analysis), FusionSeq missed one true fusion (*RPS6KB1*-*TMEM49*) because it was supported by only one mate pair. In contrast, TopHat-Fusion found this fusion because it was supported by three mate pairs from TopHat-Fusion's alignment algorithm: one mate pair contains a read that spans a splice junction, and the other contains a read that spans a fusion point. These spliced alignments are not found by Bowtie or ELAND. With this spliced mapping capability, TopHat-Fusion will be expected to have higher sensitivity than those based on non-gapped aligners. When the minimum number of mate pairs is reduced to 1, FusionSeq found all three known fusions at the expense of increased running time (9 hours versus just over 2 hours) and a large increase in the number of candidate fusions reported (32,646 versus 5,649).

Next, we ran all of FusionSeq's filters except two (PCR filter and annotation consistency filter) that would otherwise eliminate two of the true fusions. FusionSeq reported 14,510 gene fusions (Additional file 6), compared to just 14 fusions reported by TopHat-Fusion (Additional file 7), where both found the three known fusions. Among those fusions reported by FusionSeq, 13,631 and 276 were classified as inter-chromosomal and intra-chromosomal, respectively. When we used all of FusionSeq's filters, it reported 763 candidate fusions that include only one of the three known fusions.

FusionSeq reports three scores for each transcript: SPER (normalized number of inter-transcript paired-end reads), DASPER (difference between observed and expected SPER), and RESPER (ratio of observed SPER to the average of all SPERs). Because RESPER is proportional to SPER in the same data, we used SPER and DASPER to control the number of fusion candidates: *ARFGEF2*-*SULF2 *(SPER, 1.289452; DASPER, 1.279144), *BCAS4*-*BCAS3 *(0.483544, 0.482379), and *RPS6KB1*-*TMEM49 *(0.161181, 0.133692). First, we used SPER of 0.161181 and DASPER of 0.133692 to find the minimum set of fusion candidates that include the three known gene fusions. This reduced the number of candidates from 14,510 to 11,774. Second, we used the SPER and DASPER values from *ARFGEF2*-*SULF2 *and *BCAS4*-*BCAS3*, which resulted in 1,269 and 512 predicted fusions, respectively.

We next compared TopHat-Fusion with deFuse (0.4.2) [27]. deFuse maps read pairs against the genome and against cDNA sequences using Bowtie, and then uses discordantly mapped mate pairs to find candidate regions where fusion break points may lie. This allows detection of break points at base-pair resolution, similar to TopHat-Fusion. After collecting sequences around fusion points, it maps them against the genome, cDNAs, and expressed sequence tags using BLAT; this step dominates the run time.

Using two data sets - MCF7 and SKBR3 - we ran both TopHat-Fusion and deFuse using the following matched parameters: one minimum spanning read, two supporting mate pairs, and 13 bp as the anchor length. For the MCF7 cell line, both programs found the three known fusion transcripts. For the SKBR3 cell line, both programs found the same seven fusions out of nine previously reported fusion transcripts (one known fusion, *CSE1L*-ENSG00000236127, was not considered because ENSG00000236127 has been removed from the recent Ensembl database). Both programs missed two fusion transcripts: *DHX35*-*ITCH *and *NFS1*-*PREX1*. However, TopHat-Fusion had far fewer false positives: it predicted 42 fusions in total, while deFuse predicted 1,670 (Additional files 7, 8 and 9).

Table 5 shows the number of spanning reads and supporting pairs detected by TopHat-Fusion and deFuse, respectively, for ten known fusions in SKBR3 and MCF7. The numbers are similar in both programs for the known fusion transcripts. Considering the fact TopHat-Fusion's mapping step does not use annotations while deFuse does, this result illustrates that TopHat-Fusion can be highly sensitive without relying on annotations. Finally, we noted that TopHat-Fusion was approximately three times faster: for the SKBR3 cell line, it took 7 hours, while deFuse took 22 hours, both using the same eight-core computer.

**Table 5:** Comparisons of TopHat-Fusion and deFuse for SKBR3 and MCF7 cell lines

			TopHat-Fusion		deFuse	
Sample ID	Fusion genes (left-right)	Chromosomes (left-right)	Spanning reads	Spanning pairs	Spanning reads	Spanning pairs
SKBR3	*TATDN1*-*GSDMB*	8-17	311	555	322	95
SKBR3	*RARA*-*PKIA*	17-8	1	5	1	4
SKBR3	*ANKHD1*-*PCDH1*	5-5	4	15	5	11
SKBR3	*CCDC85C*-*SETD3*	14-14	5	6	6	3
SKBR3	*SUMF1*-*LRRFIP2*	3-3	3	12	5	12
SKBR3	*WDR67*-*ZNF704*	8-8	3	3	3	2
SKBR3	*CYTH1*-*EIF3H*	17-8	18	37	16	27
MCF7	*BCAS4*-*BCAS3*	20-17	105	284	106	105
MCF7	*ARFGEF2*-*SULF2*	20-20	17	20	17	12
MCF7	*RPS6KB1*-*TMEM49*	17-17	4	3	6	2

Comparisons of the number of spanning reads and mate pairs reported by TopHat-Fusion and deFuse for ten previously reported fusion transcripts in the SKBR3 and MCF7 sample data.

Unlike FusionSeq and deFuse (as well as other fusion-finding programs), one of the most powerful features in TopHat-Fusion is its ability to map reads across introns, indels, and fusion points in an efficient way and report the alignments in a modified SAM (Sequence Alignment/Map) format [28].

## Conclusions

Unlike previous approaches based on discordantly mapping paired reads and known gene annotations, TopHat-Fusion can find either individual or paired reads that span gene fusions, and it runs independently of known genes. These capabilities increase its sensitivity and allow it to find fusions that include novel genes and novel splice variants of known genes. In experiments using multiple cell lines from previous studies, TopHat-Fusion identified 34 of 38 previously known fusions. It also found 61 fusion genes not previously reported in those data, each of which had solid support from multiple reads or pairs of reads.

## Materials and methods

The first step in analysis of an RNA-seq data set is to align (map) the reads to the genome, which is complicated by the presence of introns. Because introns can be very long, particularly in mammalian genomes, the alignment program must be capable of aligning a read in two or more pieces that can be widely separated on a chromosome. The size of RNA-seq data sets, numbering in the tens of millions or even hundreds of millions of reads, demands that spliced alignment programs also be very efficient. The TopHat program achieves efficiency primarily through the use of the Bowtie aligner [13], an extremely fast and memory-efficient program for aligning unspliced reads to the genome. TopHat uses Bowtie to find all reads that align entirely within exons, and creates a set of partial exons from these alignments. It then creates hypothetical intron boundaries between the partial exons, and uses Bowtie to re-align the initially unmapped (IUM) reads and find those that define introns.

**Figure 2:**
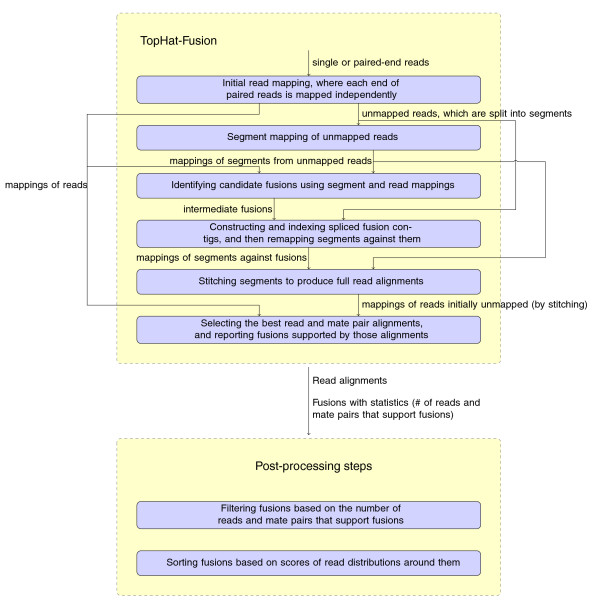
**TopHat-Fusion pipeline**. TopHat-Fusion consists of two main modules: (1) finding candidate fusions and aligning reads across them; and (2) filtering out false fusions using a series of post-processing routines.

**Figure 3:**
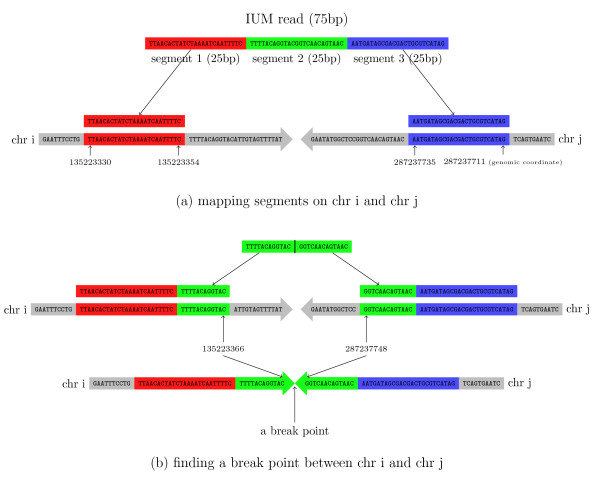
**Aligning a read that spans a fusion point**. **(a) **An initially unmapped read of 75 bp is split into three segments of 25 bp, each of which is mapped separately. As shown here, the left (red) and right (blue) segments are mapped to two different chromosomes, i and j. **(b) **The unmapped green segment is used to find the precise fusion point between i and j. This is done by aligning the green segment to the sequences just to the right of the red segment on chromosome i and just to the left of the blue segment on chromosome j.

TopHat-Fusion implements several major changes to the original TopHat algorithm, all designed to enable discovery of fusion transcripts (Figure 2). After identifying the set of IUM reads, it splits each read into multiple 25-bp pieces, with the final segment being 25 bp or longer; for example, an 80-bp read will be split into three segments of length 25, 25, and 30 (Figure 3).

The algorithm then uses Bowtie to map the 25-bp segments to the genome. For normal transcripts, the TopHat algorithm requires that segments must align in a pattern consistent with introns; that is, the segments may be separated by a user-defined maximum intron length, and they must align in the same orientation along the same chromosome. For fusion transcripts, TopHat-Fusion relaxes both these constraints, allowing it to detect fusions across chromosomes as well as fusions caused by inversions.

Following the mapping step, we filter out candidate fusion events involving multi-copy genes or other repetitive sequences, on the assumption that these sequences cause mapping artifacts. However, some multi-mapped reads (reads that align to multiple locations) might correspond to genuine fusions: for example, in Kinsella *et al. *[19], the known fusion genes *HOMEZ*-*MYH6 *and *KIAA1267*-*ARL17A *were supported by 2 and 11 multi-mapped read pairs, respectively. Therefore, instead of eliminating all multi-mapped reads, we impose an upper bound *M *(default M = 2) on the number of mappings per read. If a read or a pair of reads has M or fewer multi-mappings, then all mappings for that read are considered. Reads with > M mappings are discarded.

To further reduce the likelihood of false positives, we require that each read mapping across a fusion point have at least 13 bases matching on both sides of the fusion, with no more than two mismatches. We consider alignments to be fusion candidates when the two 'sides' of the event either (a) reside on different chromosomes or (b) reside on the same chromosome and are separated by at least 100,000 bp. The latter are the results of intra-chromosomal rearrangements or possibly read-through transcription events. We chose the 100,000-bp minimum distance as a compromise that allows TopHat-Fusion to detect intra-chromosomal rearrangements while excluding most but not all read-through transcripts. Intra-chromosomal fusions may also include inversions.

**Figure 4:**
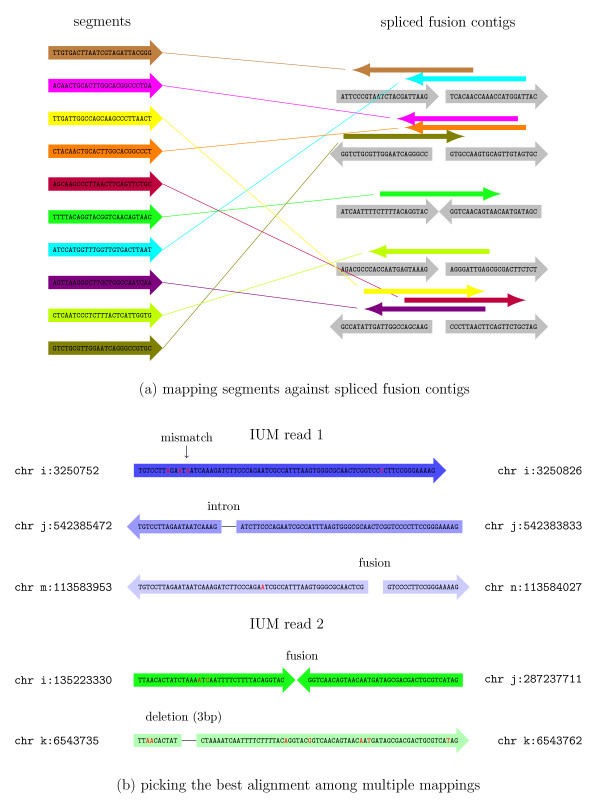
**Mapping against fusion points and selecting best read alignments**. **(a) **Bowtie is used to align all segments from the initially unmapped (IUM) reads against spliced fusion contigs, shown in gray on the right. For example, the brown read on the top left aligns to the first spliced fusion contig on the top right. **(b) **IUM reads 1 and 2 each have multiple alignments. Read 1 has a gap-free alignment, shown in dark blue, which is preferred over the other two alignments shown in lighter shades of blue. The gap-free alignment with three mismatches is preferred over the fusion alignment with one mismatch. If all alignments have gaps and mismatches, then the algorithm prefers those with fewer mismatches, as shown by the dark green alignment for IUM read 2. Full details of the scoring function that determines these preferences are described in the Materials and methods.

As shown in Figure 3a, after splitting an IUM read into three segments, the first and last segments might be mapped to two different chromosomes. Once this pattern of alignment is detected, the algorithm uses the three segments from the IUM read to find the fusion point. After finding the precise location, the segments are re-aligned, moving inward from the left and right boundaries of the original DNA fragment. The resulting mappings are combined together to give full read alignments. For this re-mapping step, TopHat-Fusion extracts 22 bp immediately flanking each fusion point and concatenates them to create 44-bp 'spliced fusion contigs' (Figure 4a). It then creates a Bowtie index (using the bowtie-build program [13]) from the spliced contigs. Using this index, it runs Bowtie to align all the segments of all IUM reads against the spliced fusion contigs. For a 25-bp segment to be mapped to a 44-bp contig, it has to span the fusion point by at least 3 bp. (For more details, see Additional files 10, 11 and 12.)

After stitching together the segment mappings to produce full alignments, we collect those reads that have at least one alignment spanning the entire read. We then choose the best alignment for each read using a heuristic scoring function, defined below. We assign penalties for alignments that span introns (-2), indels (-4), or fusions (-4). For each potential fusion, we require that spanning reads have at least 13 bp aligned on both sides of the fusion point. (This requirement alone eliminates many false positives.) After applying the penalties, if a read has more than one alignment with the same minimum penalty score, then the read with the fewest mismatches is selected. For example, in Figure 4b, IUM read 1 (in blue) is aligned to three different locations: (1) chromosome *i *with no gap, (2) chromosome *j *where it spans an intron, and (3) a fusion contig formed between chromosome *m *and chromosome *n*. Our scoring function prefers (1), followed by (2), and by (3). For IUM read 2 (Figure 4b, in green), we have two alignments: (1) a fusion formed between chromosome *i *and chromosome *j*, and (2) an alignment to chromosome *k *with a small deletion. These two alignments both incur the same penalty, but we select (1) because it has fewer mismatches.

We imposed further filters for each data set: (1) in the breast cancer cell lines (BT474, SKBR3, KPL4, MCF7), we required two supporting pairs and the sum of spanning reads and supporting pairs to be at least 5; (2) in the VCaP paired-end reads, we required the sum of spanning reads and supporting pairs to be at least 10; (3) in the UHR paired-end reads, we required (i) three spanning reads and two supporting pairs or (ii) the sum of spanning reads and supporting pairs to be at least 10; and (4) in the UHR single-end reads, we required two spanning reads. These numbers were determined empirically using known fusions as a quality control. All candidates that fail to satisfy these filters were eliminated.

In order to remove false positive fusions caused by repeats, we extract the two 23-base sequences spanning each fusion point and then map them against the entire human genome. We convert the resulting alignments into a list of pairs (chromosome name, genomic coordinate - for example, chr14:374384). For each 23-mer adjacent to a fusion point, we test to determine if the other 23-mer occurs within 100,000 bp on the same chromosome. If so, then it is likely a repeat and we eliminate the fusion candidate. We further require that at least one side of a fusion contains an annotated gene (based on known genes from RefSeq), otherwise the fusion is filtered out. These steps alone reduced the number of fusion candidates in our experiments from 10^5 ^to just a few hundred.

**Figure 5:**
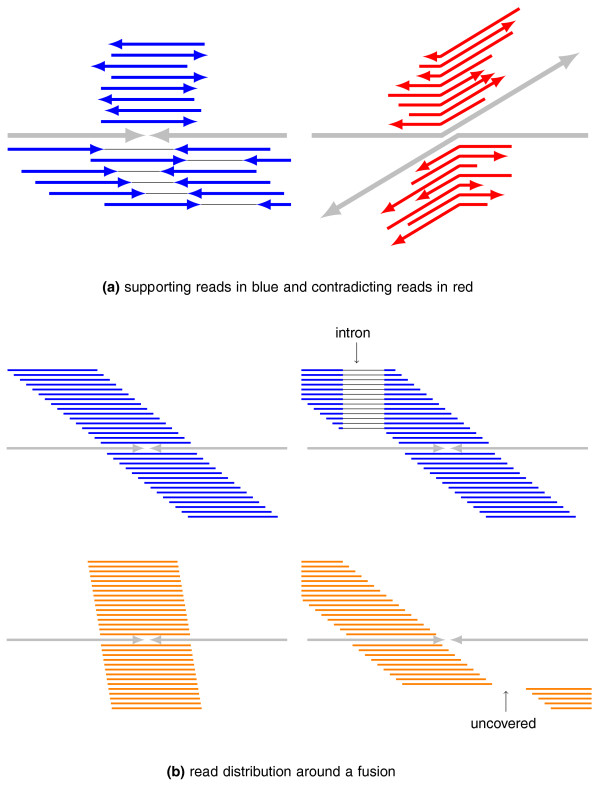
**Supporting and contradicting evidence for fusion transcripts**. **(a) **Given a fusion point and the chromosomes (gray) spanning it, single-end and paired-end reads (blue) support the fusion. Other reads (red) contradict the fusion by mapping entirely to either of the two chromosomes. **(b) **TopHat-Fusion prefers reads that uniformly cover a 600-bp window centered in any fusion point. On the upper left, blue reads cover the entire window. On the lower left, red reads cover only a narrow window around the fusion. On the lower right, reads do not cover part of the 600-bp window. The cases shown in orange will be rejected by TopHat-Fusion.

**Figure 6:**
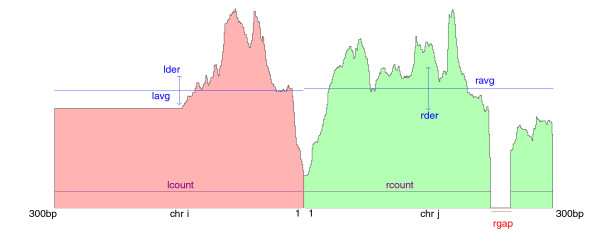
**TopHat-Fusion's scoring scheme of read distributions**. A scoring scheme of how well distributed reads are around a fusion point; these result scores are used to sort the list of candidate fusions. Variables are defined in the main text.

As reported in Edgren *et al. *[12], true fusion transcripts have reads mapping uniformly in a wide window across the fusion point, whereas false positive fusions are narrowly covered. Using this idea, TopHat-Fusion examines a 600-bp window around each fusion (300-bp each side), and rejects fusion candidates for which the reads fail to cover this window (Figure 5b). The final process is to sort fusions based on how well-distributed the reads are (Figure 6). The scoring scheme prefers alignments that have no gaps (or small gaps) and uniform depth.

Even with strict parameters for the initial alignment, many of the segments will map to multiple locations, which can make it appear that a read spans two chromosomes. Thus the algorithm may find large numbers of false positives, primarily due to the presence of millions of repetitive sequences in the human genome. Even after filtering to choose the best alignment per read, the experiments reported here yielded initial sets of about 400,000 and 135,000 fusion gene candidates from the breast cancer (BT474, SKBR3, KPL4, MCF7) and prostate cancer (VCaP) cell lines, respectively. The additional filtering steps eliminated the vast majority of these false positives, reducing the output to 76 and 19 fusion candidates, respectively, all of which have strong supporting evidence (Tables 2 and 3).

The scoring function used to rank fusion candidates uses the number of paired reads in which the reads map on either side of the fusion point in a consistent orientation (Figure 5a) as well as the number of reads in conflict with the fusion point. Conflicting reads align entirely to either of the two chromosomes and span the point at which the chromosome break should occur (Figure 5b).

The overall fusion score is computed as:

$$\begin{array}{c} \text{score}=\text{lcount}+\text{rcount}+\text{min}\left(\text{max}\text{\_}\text{avg},\;\text{lavg}\right)+\text{min}\left(\text{max}\text{\_}\text{avg},\;\text{ravg}\right)\\ \;\;\;\;\;\left|\text{lcount}-\text{rcount}\right|-\text{min}\left(\text{max}\text{\_}\text{avg},\;|\text{lavg}-\text{ravg}|\right)\\ \;\;\;\;\;\left(\text{lgap}+\text{rgap}\right)-\left(\text{lder}+\text{rder}\right)\times \text{max}\text{\_}\text{avg}+\text{rate}\\ \;\;\;\;\;\text{min}\left(\text{1}000,\;\text{dist}\right) \end{array}$$

where lcount is the number of bases covered in a 300-bp window on the left (Figure 6), lavg is the average read coverage on the left, max_avg is 300, lgap is the length of any gap on the left, rate is the ratio between the number of supporting mate pairs and the number of contradicting reads, |lavg - ravg| is a penalty for expression differences on either side of the fusion, and dist is the sum of distances between each end of a pair and a fusion. (For single-end reads, the rate uses spanning reads rather than mate pairs.) The variance in coverage lder is:

$$\text{lder}=\text{square root of sum of }{\left(\left(\text{lavg}-\text{ldept}{\text{h}}_{\text{n}}\right)∕\text{lavg}\right)}^{2}∕\text{lwindow from n}=\text{1}\;\text{to}\;\text{n}=\text{lwindow}$$

where lwindow is the size of the left window (300 bp).

TopHat-Fusion outputs alignments of singleton reads and paired-end reads mapped across fusion points in SAM format [28], enabling further downstream analyses [29], such as transcript assembly and differential gene expression. The parameters in the filtering steps can be changed as needed for a particular data set.

## Abbreviations

bp: base pair; DASPER: difference between observed and expected SPER; IUM: initially unmapped; RESPER: ratio of observed SPER to the average of all SPERs; SAM: Sequence Alignment/Map; SPER: supportive paired-end reads; UHR: universal human reference.

## Authors' contributions

DK developed the TopHat-Fusion algorithms, performed the analysis and discussed the results, implemented TopHat-Fusion and wrote the manuscript. SLS developed the TopHat-Fusion algorithms, performed the analysis and discussed the results, and wrote the manuscript. All authors have read and approved the manuscript for publication.

## Supplementary Material

Additional file 1 **Table S1 - 76 candidate fusions including multiple fusion points in the breast cancer cell lines**. Additional details for the 76 fusions detected by TopHat-Fusion in the breast cancer cell lines (BT474, SKBR3, KPL4, MCF7). Some of the genes contain multiple fusion points, presumably due to alternative splicing. Click here for file

Additional file 2 **Table S2 - 19 candidate fusions including multiple fusion points in the prostate cancer cell line**. Nineteen fusion genes detected by TopHat-Fusion in a prostate cancer cell line (VCaP), including several with multiple fusion points due to alternative splicing. Click here for file

Additional file 3 **Figure S1 - read distributions around *BCR*-*ABL1 *fusion for single-end and paired-end reads**. This figure shows read distributions around the *BCR*-*ABL1 *fusion gene in Universal Human Reference (UHR) data. **(a) **The read distribution for single-end reads (100 bp or less). **(b) **Read distribution for paired-end reads (50 bp) from 300-bp fragments. Coverage was similar with either data set. Click here for file

Additional file 4 **Table S3 - the top 20 fusion candidates reported by TopHat-Fusion in the UHR data**. The top 20 fusion genes from the Universal Human Reference (UHR) data found by TopHat-Fusion, sorted by the scoring scheme described in Figure 6. Single- and paired-end reads were used separately in order to compare TopHat's ability to find fusions using only single-end reads. Click here for file

Additional file 5 **Table S4 - 45 fusion candidates reported by TopHat-Fusion in Illumina Body Map 2.0 data**. Using two samples (testes and thyroid) from Illumina Body Map 2.0 data, TopHat-Fusion reports 45 fusions. Click here for file

Additional file 6 **List of 14,510 fusion candidates reported by FusionSeq for MCF7 sample data**. Click here for file

Additional file 7 **Table S5 - 42 fusion candidates reported by TopHat-Fusion in SKBR3 and MCF7 cell lines**. Twenty-eight and fourteen candidate fusions are reported in SKBR3 and MCF7 samples, respectively, when the filtering parameters are changed to one spanning read and two supporting mate pairs. Click here for file

Additional file 8 **List of 275 fusion candidates reported by deFuse in MCF7 sample data**. Click here for file

Additional file 9 **List of 1,395 fusion candidates reported by deFuse in SKBR3 sample data**. Click here for file

Additional file 10 **Supplementary methods**. Click here for file

Additional file 11 **Figure S2 - Finding fusions using two segments and partner reads in paired-end reads**. **(a) **TopHat allows one to three mismatches when mapping segments using Bowtie, which enables segments to be mapped even if a few bases cross a fusion point (the last two bases of the red segment, GG). These two segments, mapped to two different chromosomes, are used to identify a fusion point. **(b) **For paired-end reads, the mapped position of the partner read is used to narrow down the range of a fusion point. The second segment (shown in green) cannot be mapped because it spans a fusion point. Here, its partner read is mapped and the fusion point is likely to be located within the inner mate distance ± standard deviation of the left genomic coordinate of the partner read. TopHat-Fusion is able to use this relatively small range to efficiently map the right part of the second segment to the right side of a fusion (case 2). The left part of the second segment is aligned to the right side of the mapped first segment (case 3). Click here for file

Additional file 12 **Figure S3 - stitching segments to produce a full read alignment**. **(a) **The segment in the third row for segment 1 and the one in the first row for segment 2 are connected because they are on the same chromosome (i) in the forward direction and with adjacent coordinates. These are then matched to the second row in segment 3 and glued together, producing the full-length read alignment at the bottom. **(b) **TopHat-Fusion tries to connect the segment in the second row for segment 1 with segments in the first and second rows for segment 2, but neither succeeds. Case 1 would require two fusion points in the same read, and case 2 cannot be fused with consistent coordinates. **(c) **Attempts to connect the segment in the second row for segment 2 with the one in the first row in segment 3: in case 3, there is no intron available, there is no fusion in case 4, and case 5 would require more than one fusion. Click here for file
