# Supplementary material for: TopHat-Fusion: an algorithm for discovery of novel fusion transcripts
Source: Genome Biol. 2011 Aug 11;12(8):R72. doi: 10.1186/gb-2011-12-8-r72 (PMC3245612; doi:10.1186/gb-2011-12-8-r72)
Supplement: Additional file 1 — Table S1 - 76 candidate fusions including multiple fusion points in the breast cancer cell lines. Additional details for the 76 fusions detected by TopHat-Fusion in the breast cancer cell lines (BT474, SKBR3, KPL4, MCF7). Some of the genes contain multiple fusion points, presumably due to alternative splicing. [file gb-2011-12-8-r72-S1.PDF]

SAMPLE ID	Fusion genes (left-right)	Chromosomes (left-right)	5′ position	3′ position	Spanning reads	Spanning pairs
BT474	TRPC4AP-MRPL45	20-17	33665850	36476499	2	9
BT474	TRPC4AP-MRPL45	20-17	33665850	36478006	2	13
BT474	ENSG00000141232-SYNRG	17-17	48943418	35880750	26	47
SKBR3	TATDN1-GSDMB	8-17	125551165	38062234	4	80
SKBR3	TATDN1-GSDMB	8-17	125551167	38066175	14	101
SKBR3	TATDN1-GSDMB	8-17	125551262	38062234	76	204
SKBR3	TATDN1-GSDMB	8-17	125551264	38062522	38	126
SKBR3	TATDN1-GSDMB	8-17	125551264	38066175	311	555
SKBR3	TATDN1-GSDMB	8-17	125551265	38063239	3	76
BT474	THRA-SKAP1	17-17	38243102	46384689	28	46
BT474	THRA-SKAP1	17-17	38243103	46371085	2	63
BT474	THRA-SKAP1	17-17	38243103	46371706	85	126
MCF7	BCAS4-BCAS3	20-17	49411707	59430946	10	15
MCF7	BCAS4-BCAS3	20-17	49411707	59445685	105	284
BT474	ACACA-STAC2	17-17	35479452	37374425	57	59
BT474	STX16-RAE1	20-20	57227142	55929087	6	24
BT474	MED1-ENSG00000167107	17-17	37595419	48548386	10	12
MCF7	ENSG00000254868-FOXA1	14-14	38184710	38061534	2	22
SKBR3	ANKHD1-PCDH1	5-5	139825557	141234002	4	15
BT474	ZMYND8-CEP250	20-20	45852972	34078459	10	53
BT474	ENSG00000153207-NAAA	1-4	247094879	76846963	10	42
SKBR3	SUMF1-LRRFIP2	3-3	4418012	37170638	3	12
SKBR3	SUMF1-LRRFIP2	3-3	4452547	37170638	3	8
KPL4	BSG-NFIX	19-19	580779	13133337	2	22
KPL4	BSG-NFIX	19-19	580779	13135832	12	27
BT474	VAPB-IKZF3	20-17	56964570	37944628	3	10
BT474	VAPB-IKZF3	20-17	56964572	37934019	19	53
BT474	VAPB-IKZF3	20-17	56964574	37922743	4	14
BT474	ENSG00000150672-HFM1	11-1	85195025	91853144	2	10
SKBR3	CSE1L-ENSG00000236127	20-20	47688988	47956855	13	31
MCF7	RSBN1-AP4B1	1-1	114354329	114442495	6	7
BT474	MED13-BCAS3	17-17	60129899	59469335	3	14
MCF7	ARFGEF2-SULF2	20-20	47538545	46365686	17	20
BT474	HFM1-ENSG00000198744	1-1	91853132	570112	1	47
BT474	HFM1-ENSG00000198744	1-1	91853140	570103	5	44
KPL4	HFM1-ENSG00000198744	1-1	91853140	570103	2	5
BT474	HFM1-ENSG00000225630	1-1	91853144	565937	2	43
KPL4	MUC20-ENSG00000249796	3-3	195456606	195352198	13	46
KPL4	MUC20-ENSG00000236833	3-3	195456612	197391649	8	15
MCF7	RPS6KB1-TMEM49	17-17	57992061	57917126	4	3
SKBR3	WDR67-ZNF704	8-8	124096577	81733851	3	3
BT474	CPNE1-PI3	20-20	34243123	43804501	2	6
BT474	ENSG00000229344-RYR2	1-1	568361	237766339	1	19
BT474	ENSG00000229344-RYR2	1-1	568365	237766476	2	19
BT474	LAMP1-MCF2L	13-13	113951808	113718616	2	6
MCF7	SULF2-ENSG00000171940	20-20	46415145	52210297	22	48
MCF7	SULF2-ENSG00000171940	20-20	46415146	52210647	11	32
BT474	WBSCR17-FBXL20	7-17	70958325	37557612	2	8
MCF7	ENSG00000224738-TMEM49	17-17	57184949	57915653	5	6
MCF7	ANKRD30BL-RPS23	2-5	133012791	81574161	2	6
BT474	ENSG00000251948-SLCO5A1	19-8	24184149	70602608	2	6
SKBR3	ENSG00000251948-SLCO5A1	19-8	24184150	70602607	1	4
BT474	GLB1-CMTM7	3-3	33055545	32483333	2	6
KPL4	EEF1DP3-FRY	13-13	32520314	32652967	2	4
MCF7	PAPOLA-AK7	14-14	96968936	96904171	3	3
BT474	ZNF185-GABRA3	X-X	152114004	151468336	2	3
KPL4	PPP1R12A-SEPT10	12-2	80211173	110343414	3	8
BT474	SKA2-MYO19	17-17	57232490	34863349	5	12
BT474	SKA2-MYO19	17-17	57232490	34863761	6	14
MCF7	LRP1B-PLXDC1	2-17	142237963	37265642	2	5

SAMPLE ID	Fusion genes (left-right)	Chromosomes (left-right)	5′ position	3′ position	Spanning reads	Spanning pairs
BT474	NDUFB8-TUBD1	10-17	102289117	57962592	1	49
BT474	ENSG00000225630-NOTCH2NL	1-1	565870	145277319	1	18
SKBR3	CYTH1-EIF3H	17-8	76778281	117738408	1	10
SKBR3	CYTH1-EIF3H	17-8	76778283	117671218	1	14
SKBR3	CYTH1-EIF3H	17-8	76778283	117768257	18	37
BT474	PSMD3-ENSG00000237973	17-1	38151673	566925	1	12
BT474	STARD3-DOK5	17-20	37793479	53259992	2	10
BT474	DIDO1-TTI1	20-20	61569147	36634798	1	10
BT474	RAB22A-MYO9B	20-19	56886176	17256205	8	20
KPL4	PCBD2-ENSG00000240967	5-5	134259840	99382129	1	32
SKBR3	RARA-PKIA	17-8	38465534	79485042	9	19
SKBR3	RARA-PKIA	17-8	38465535	79479713	1	11
SKBR3	RARA-PKIA	17-8	38465535	79510590	1	5
BT474	MED1-STXBP4	17-17	37607288	53218672	13	11
KPL4	ENSG00000173436-ENSG00000224237	1-3	19923605	27256479	1	5
BT474	RNF6-FOXO1	13-13	26795971	41192773	1	8
SKBR3	RNF6-FOXO1	13-13	26795971	41192773	2	13
SKBR3	BAT1-ENSG00000254406	6-11	31499072	119692419	2	30
BT474	ENSG00000185261-PCBD2	5-5	93904985	134259811	1	19
BT474	PCBD2-ANKRD30BL	5-2	134261791	133012976	2	7
SKBR3	PCBD2-ANKRD30BL	5-2	134263179	133012790	1	5
BT474	ENSG00000225630-MTRNR2L8	1-11	565457	10530147	1	35
BT474	ENSG00000225630-MTRNR2L8	1-11	565485	10530145	2	36
BT474	PCBD2-ENSG00000251948	5-19	134260431	24184146	2	6
BT474	ANKRD30BL-ENSG00000237973	2-1	133012085	567103	2	8
KPL4	ENSG00000225972-HSP90AB1	1-6	564639	44220780	1	7
BT474	MTIF2-ENSG00000228826	2-1	55470625	121244943	1	11
BT474	ENSG00000224905-PCBD2	21-5	15457432	134263223	2	7
BT474	RPS6KB1-SNF8	17-17	57970686	47021335	48	57
BT474	RPS6KB1-SNF8	17-17	57971285	47021335	1	32
BT474	MTRNR2L8-PCBD2	11-5	10530146	134263156	1	6
BT474	RPL23-ENSG00000225630	17-1	37009355	565697	3	19
BT474	MTRNR2L2-PCBD2	5-5	79946288	134259832	1	5
BT474	MTRNR2L2-PCBD2	5-5	79946305	134260456	2	5
BT474	MTRNR2L2-PCBD2	5-5	79946847	134262438	1	5
BT474	MTRNR2L2-PCBD2	5-5	79946851	134263739	1	5
SKBR3	PCBD2-ENSG00000239776	5-12	134263289	127650986	2	3
SKBR3	ENSG00000240409-PCBD2	1-5	569010	134259295	1	4
SKBR3	ENSG00000240409-PCBD2	1-5	569018	134260144	2	5
SKBR3	ENSG00000240409-PCBD2	1-5	569021	134263951	1	4
SKBR3	ENSG00000240409-PCBD2	1-5	569029	134259827	1	4
BT474	ENSG00000239776-MTRNR2L2	12-5	127650981	79946277	2	3
BT474	JAK2-TCF3	9-19	5112849	1610500	1	46
KPL4	NOTCH1-NUP214	9-9	139438475	134062675	3	5
BT474	MTRNR2L8-ENSG00000240215	11-9	10530594	33657801	4	4
BT474	MTRNR2L8-AKAP6	11-14	10530179	32953468	1	5
BT474	ENSG00000230916-PCBD2	X-5	125606246	134263219	1	5
BT474	ENSG00000226505-MRPL36	2-5	70329650	1799907	13	36
MCF7	ENSG00000226505-MRPL36	2-5	70329650	1799907	5	20
SKBR3	CCDC85C-SETD3	14-14	100002351	99880270	5	6
BT474	RPL23-ENSG00000230406	17-2	37009955	222457168	109	5
