# Supplementary material for: TopHat-Fusion: an algorithm for discovery of novel fusion transcripts
Source: Genome Biol. 2011 Aug 11;12(8):R72. doi: 10.1186/gb-2011-12-8-r72 (PMC3245612; doi:10.1186/gb-2011-12-8-r72)
Supplement: Additional file 2 — Table S2 - 19 candidate fusions including multiple fusion points in the prostate cancer cell line. Nineteen fusion genes detected by TopHat-Fusion in a prostate cancer cell line (VCaP), including several with multiple fusion points due to alternative splicing. [file gb-2011-12-8-r72-S2.PDF]

SAMPLE ID	Fusion genes (left-right)	Chromosomes (left-right)	5′ position	3′ position	Spanning reads	Spanning pairs
VCaP	ZDHHC7-ABCB9	16-12	85022367	123451311	2	42
VCaP	ZDHHC7-ABCB9	16-12	85023908	123444867	13	69
VCaP	ZDHHC7-ABCB9	16-12	85023908	123451311	1	43
VCaP	TMPRSS2-ERG	21-21	42879875	39817542	7	285
VCaP	TMPRSS2-ERG	21-21	42880006	39817542	100	505
VCaP	HJURP-EIF4E2	2-2	234749254	233421125	3	9
VCaP	VWA2-PRKCH	10-14	116008521	61909826	1	10
VCaP	RGS3-PRKAR1B	9-7	116299194	697377	1	9
VCaP	RGS3-PRKAR1B	9-7	116299195	699055	3	11
VCaP	SPOCK1-TBC1D9B	5-5	136397966	179305324	9	31
VCaP	LRP4-FBXL20	11-17	46911864	37557613	5	9
VCaP	INPP4A-HJURP	2-2	99193605	234746297	6	12
VCaP	ENSG00000125149-ENSG00000124074	16-16	67144140	67700168	2	19
VCaP	ENSG00000125149-ENSG00000159761	16-16	67144140	67700996	3	20
VCaP	NDUFV2-ENSG00000188699	18-19	9102729	53727808	1	35
VCaP	NEAT1-ENSG00000229344	11-1	65190281	568419	1	17
VCaP	ENSG00000011405-TEAD1	11-11	17229396	12883794	7	9
VCaP	USP10-ZDHHC7	16-16	84733713	85024243	1	22
VCaP	LMAN2-AP3S1	5-5	176778452	115202366	15	2
VCaP	WDR45L-ENSG00000224737	17-17	80579516	30439195	1	33
VCaP	RC3H2-RGS3	9-9	125622198	116299072	3	11
VCaP	CTNNA1-ENSG00000249026	5-5	138145895	114727795	1	12
VCaP	ENSG00000229880-IMMT	21-2	46097128	86389185	1	50
VCaP	ENSG00000214009-PCNA	X-20	45918367	5098168	1	24
