# Supplementary figures and images for: TopHat-Fusion: an algorithm for discovery of novel fusion transcripts
Source: Genome Biol. 2011 Aug 11;12(8):R72. doi: 10.1186/gb-2011-12-8-r72 (PMC3245612; doi:10.1186/gb-2011-12-8-r72)

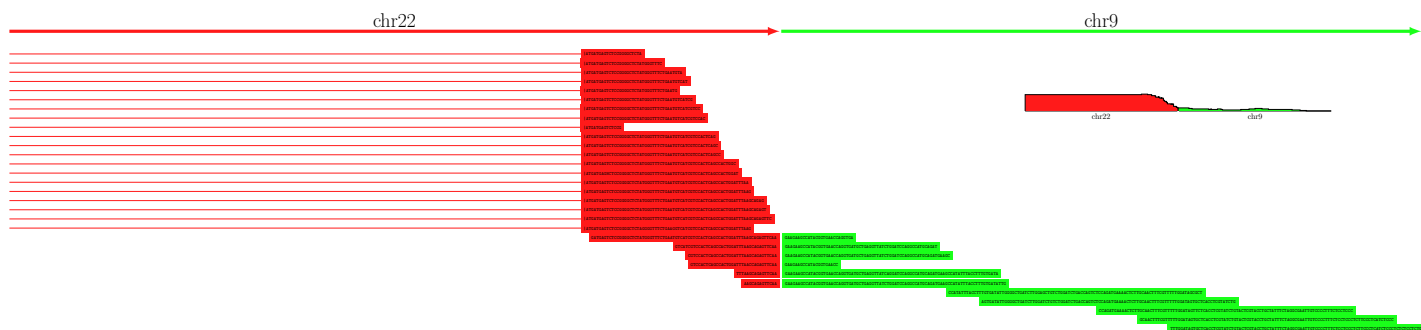

(a) BCR-ABL1 in UHR single ends

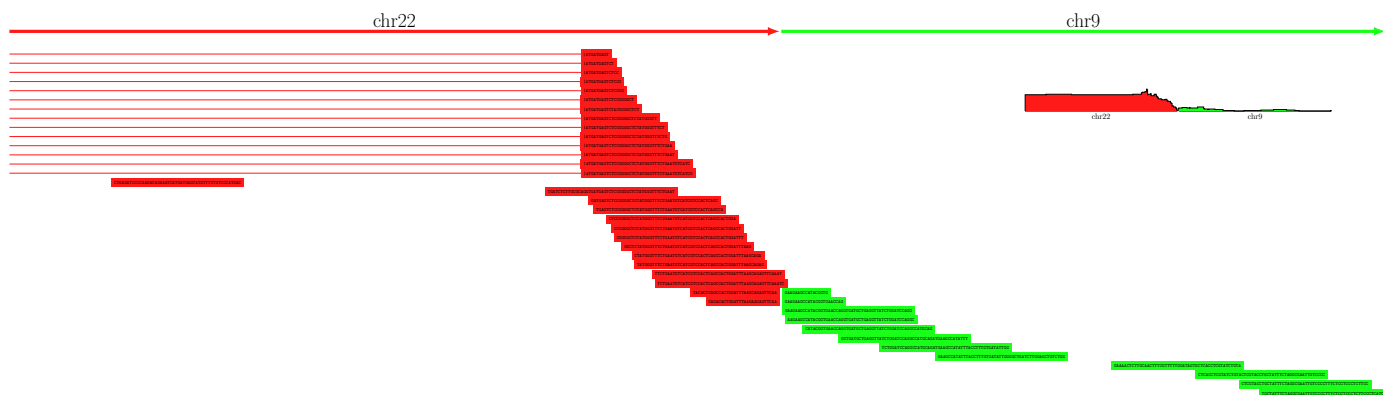

(b) BCR-ABL1 in UHR paired ends

Supplement: Additional file 3 — Figure S1 - read distributions around BCR-ABL1 fusion for single-end and paired-end reads. This figure shows read distributions around the BCR-ABL1 fusion gene in Universal Human Reference (UHR) data. (a) The read distribution for single-end reads (100 bp or less). (b) Read distribution for paired-end reads (50 bp) from 300-bp fragments. Coverage was similar with either data set. [file gb-2011-12-8-r72-S3.PDF]

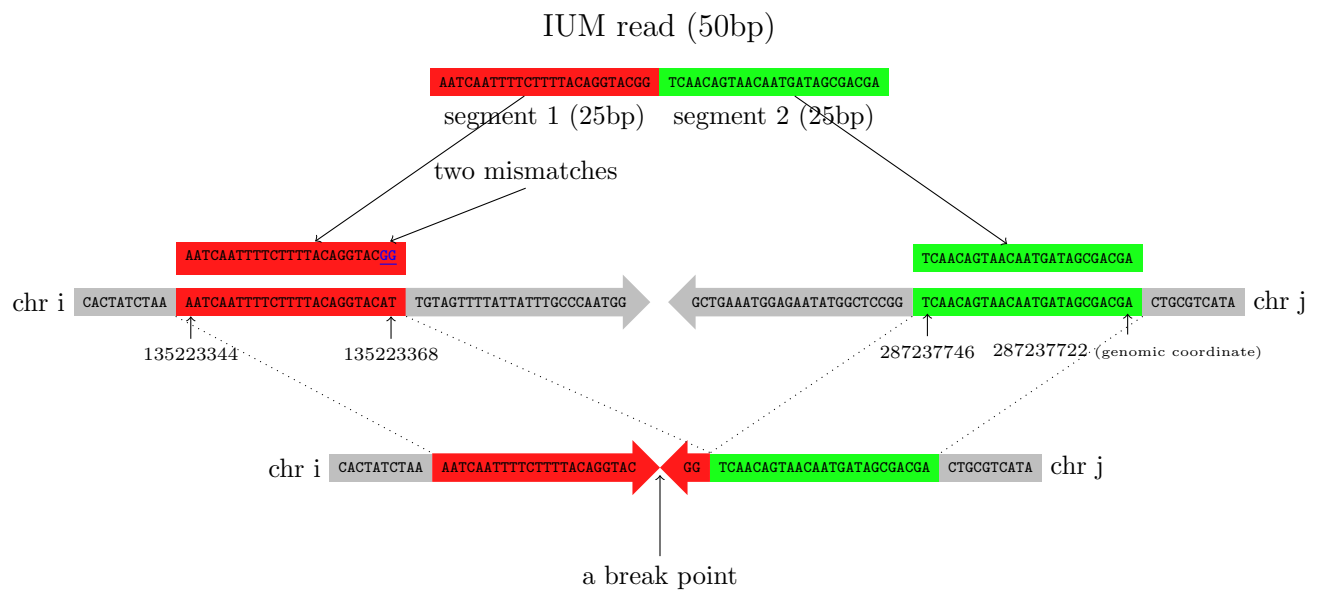

(a) finding a fusion in case of two segments

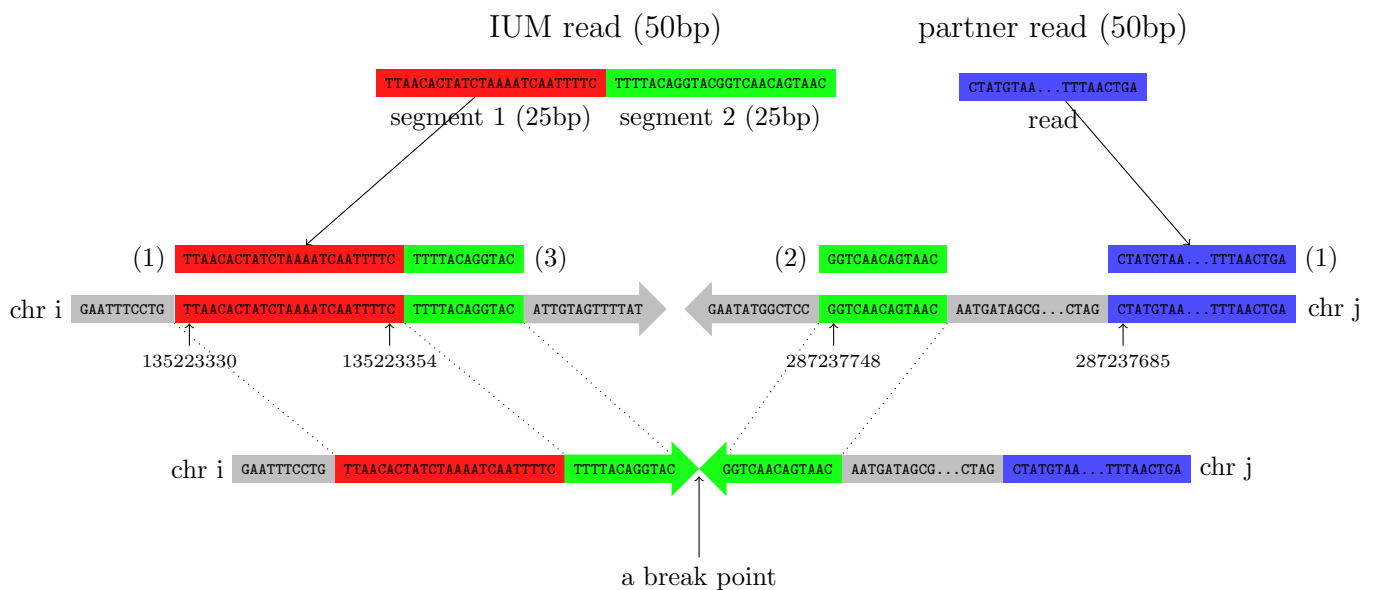

(b) finding a fusion using paired-end reads

Supplement: Additional file 11 — Figure S2 - Finding fusions using two segments and partner reads in paired-end reads. (a) TopHat allows one to three mismatches when mapping segments using Bowtie, which enables segments to be mapped even if a few bases cross a fusion point (the last two bases of the red segment, GG). These two segments, mapped to two different chromosomes, are used to identify a fusion point. (b) For paired-end reads, the mapped position of the partner read is used to narrow down the range of a fusion point. The second segment (shown in green) cannot be mapped because it spans a fusion point. Here, its partner read is mapped and the fusion point is likely to be located within the inner mate distance ± standard deviation of the left genomic coordinate of the partner read. TopHat-Fusion is able to use this relatively small range to efficiently map the right part of the second segment to the right side of a fusion (case 2). The left part of the second segment is aligned to the right side of the mapped first segment (case 3). [file gb-2011-12-8-r72-S11.PDF]
