# Supplementary material for: TopHat-Fusion: an algorithm for discovery of novel fusion transcripts
Source: Genome Biol. 2011 Aug 11;12(8):R72. doi: 10.1186/gb-2011-12-8-r72 (PMC3245612; doi:10.1186/gb-2011-12-8-r72)
Supplement: Additional file 4 — Table S3 - the top 20 fusion candidates reported by TopHat-Fusion in the UHR data. The top 20 fusion genes from the Universal Human Reference (UHR) data found by TopHat-Fusion, sorted by the scoring scheme described in Figure 6. Single- and paired-end reads were used separately in order to compare TopHat's ability to find fusions using only single-end reads. [file gb-2011-12-8-r72-S4.PDF]

Read type	Fusion genes (left-right)	Chromosomes (left-right)	5′ position	3′ position	Spanning reads	Spanning pairs
Single	GAS6-RASA3	13-13	114529968	114751268	15	
Paired	GAS6-RASA3	13-13	114529968	114751268	10	43
Paired	NEAT1-ENSG00000229344	11-1	65190276	568376	2	19
Single	BAG6-SLC44A4	6-6	31619432	31833560	3	
Paired	BAG6-SLC44A4	6-6	31619432	31833560	4	8
Paired	ENSG00000254868-FOXA1	14-14	38184710	38061534	1	9
Paired	SCARB1-UBC	12-12	125348140	125398319	1	3
Single	BCR-ABL1	22-9	23632599	133655755	6	0
Single	BCR-ABL1	22-9	23632599	133729450	3	0
Paired	BCR-ABL1	22-9	23632599	133655755	2	7
Paired	BCR-ABL1	22-9	23632599	133729450	3	10
Paired	ARFGEF2-SULF2	20-20	47538545	46365686	10	30
Single	ARFGEF2-SULF2	20-20	47538548	46365683	17	
Paired	NCL-ENSG00000240409	2-1	232319979	569028	1	19
Single	BCAS4-BCAS3	20-17	49411707	59445685	25	
Paired	BCAS4-BCAS3	20-17	49411707	59445685	13	145
Paired	CNOT10-NOL6	3-9	32794794	33472410	2	4
Paired	ENSG00000116668-ENSG00000121486	1-1	185128210	185097799	2	7
Single	NUP214-XKR3	9-22	134074401	17288972	2	
Paired	NUP214-XKR3	9-22	134074401	17288972	3	11
Single	UBB-ENSG00000237973	17-1	16284425	566987	2	
Paired	UBB-ENSG00000237973	17-1	16284425	566987	6	9
Single	SULF2-ENSG00000171940	20-20	46415145	52210297	2	
Paired	SULF2-ENSG00000171940	20-20	46415145	52210297	4	14
Single	SULF2-ENSG00000171940	20-20	46415149	52210644	2	
Paired	SULF2-ENSG00000171940	20-20	46415149	52210644	1	10
Paired	PPEF2-ENSG00000224905	4-21	76807191	15457266	1	2
Paired	NTAN1-FAM82B	16-8	15141353	87494987	1	23
Single	SEC14L1-ENSG00000243092	17-12	75158386	34372606	271	
Single	HNRNPL-SSRP1	19-11	39329532	57094346	2	
Single	MCM7-RPS24	7-10	99697710	79793622	2	
Paired	PCBD2-ENSG00000115053	5-2	134263552	232326713	1	6
