# Supplementary material for: TopHat-Fusion: an algorithm for discovery of novel fusion transcripts
Source: Genome Biol. 2011 Aug 11;12(8):R72. doi: 10.1186/gb-2011-12-8-r72 (PMC3245612; doi:10.1186/gb-2011-12-8-r72)
Supplement: Additional file 5 — Table S4 - 45 fusion candidates reported by TopHat-Fusion in Illumina Body Map 2.0 data. Using two samples (testes and thyroid) from Illumina Body Map 2.0 data, TopHat-Fusion reports 45 fusions. [file gb-2011-12-8-r72-S5.PDF]

SAMPLE ID	Fusion genes (left-right)	Chromosomes (left-right)	$5'$ position	$3'$ position	Spanning reads	Spanning pairs
thyroid	CDC14A-BCAS2	1-1	100859004	115123919	11	286
testes	CCT4-COMMD1	2-2	62115717	62227834	3	54
thyroid	FAM18A-NUBP1	16-16	10861820	10855224	1	11
thyroid	ENSG00000237676-RPL30	3-8	32677040	99057616	60	28
testes	ENSG00000237676-RPL30	3-8	32677040	99057616	14	20
thyroid	TPO-ENSG00000225630	2-1	1499764	565860	5	46
thyroid	HSP90AB1-HSP90AB2P	6-4	44218040	13338731	3	97
thyroid	HFM1-ENSG00000198744	1-1	91853140	570103	2	20
thyroid	PKP4-ENSG00000240286	2-2	159526371	210045255	2	8
thyroid	FUS-ENSG00000198744	16-1	31202289	570100	1	40
thyroid	IL6R-PSMD8	1-19	154387371	38869888	1	12
thyroid	MYL6-ENSG00000228118	12-17	56554034	69617454	3	28
thyroid	LOC729852-ENSG00000233108	7-7	7841371	8007327	8	4
thyroid	CSDE1-ENSG00000248527	1-1	115263334	569602	3	51
thyroid	ENSG00000223825-DAZAP2	2-12	203066609	51632607	2	14
thyroid	CAGE1-RPS3	6-11	7339787	75111737	3	19
thyroid	HSP90B1-LRRC28	12-15	104337084	99799716	4	28
thyroid	ENSG00000176243-CDV3	3-3	96496205	133305418	5	18
thyroid	ENSG00000176243-CDV3	3-3	96496205	133305424	2	18
thyroid	ENSG00000137807-RPLP1	15-15	69745156	69745189	4	56
testes	ENSG00000137807-RPLP1	15-15	69745156	69745189	3	43
thyroid	ENSG00000238193-HADHB	X-2	5225090	26477307	4	11
thyroid	NCRNA00182-DDX3X	X-X	73350977	41204709	2	80
thyroid	ENSG00000214047-GOLGA3	X-12	39724922	133402274	5	24
thyroid	ENSG00000214047-GOLGA3	X-12	39724922	133402288	3	20
thyroid	ENSG00000239617-RPL32	12-3	49297528	12881023	8	7
thyroid	HRH1-CHCHD4	3-3	11276358	14154604	2	3
thyroid	PCBD2-C5orf36	5-5	134259070	93906073	9	4
thyroid	PCBD2-C5orf36	5-5	134259116	93906090	2	4
thyroid	PCBD2-C5orf36	5-5	134259127	93906097	5	4
thyroid	PCBD2-C5orf36	5-5	134259170	93906077	2	4
thyroid	PCBD2-C5orf36	5-5	134259172	93906076	4	4
thyroid	PCBD2-C5orf36	5-5	134259845	93905187	2	4
thyroid	PCBD2-C5orf36	5-5	134260445	93904678	3	4
thyroid	PCBD2-C5orf36	5-5	134260508	93904686	14	4
thyroid	PCBD2-C5orf36	5-5	134260514	93904682	14	4
thyroid	ACO2-POLR3H	22-22	41921340	41922274	3	24
thyroid	ENSG00000240155-PCBD2	1-5	565016	134261777	6	65
thyroid	CD72-TESK1	9-9	35609976	35609347	1	32
thyroid	OTUD7B-ENSG00000240409	1-1	149916531	568956	2	12
thyroid	COL6A2-ZNF688	21-16	47542847	30581751	1	3
thyroid	PCBD2-TMBIM1	5-2	134261417	219144833	2	6
thyroid	ENSG00000100226-SUN2	22-22	39130173	39131271	2	98
thyroid	RPL23-ENSG00000180211	17-6	37009985	39926565	11	12
thyroid	RPL23-ENSG00000180211	17-6	37009986	39926565	4	14
thyroid	ENSG00000237973-ATP6V0B	1-1	567099	44441673	1	7
thyroid	RPLP2-ENSG00000240652	11-11	811609	107779258	3	57
thyroid	RPL37-ENSG00000241431	5-8	40835323	57500957	2	7
thyroid	ENSG00000237973-BTG2	1-1	567121	203275477	6	9
thyroid	ENSG00000248327-NOL8	4-9	166469498	95072941	4	91
thyroid	ENSG00000248827-USP7	5-16	107061668	9010410	4	91
thyroid	ENSG00000197857-RPL28	19-19	12370929	55897302	1	4
thyroid	LOC96610-ENSG00000211676	22-22	22677321	23241797	5	22
thyroid	PCBD2-TPO	5-2	134262389	1499769	1	3
testes	PCBD2-ENSG00000240967	5-5	134260002	99382129	11	2
thyroid	C3orf26-FILIP1L	3-3	99536884	99730569	2	5
thyroid	PCBD2-C5orf36	5-5	134259138	93905608	5	3
thyroid	PCBD2-C5orf36	5-5	134259224	93905741	10	3
thyroid	PCBD2-C5orf36	5-5	134259247	93905771	27	3
thyroid	PCBD2-C5orf36	5-5	134260396	93904666	4	3
thyroid	CNGB3-UBE2Q2	8-15	87672907	76170299	2	18
