# Supplementary material for: TopHat-Fusion: an algorithm for discovery of novel fusion transcripts
Source: Genome Biol. 2011 Aug 11;12(8):R72. doi: 10.1186/gb-2011-12-8-r72 (PMC3245612; doi:10.1186/gb-2011-12-8-r72)
Supplement: Additional file 7 — Table S5 - 42 fusion candidates reported by TopHat-Fusion in SKBR3 and MCF7 cell lines. Twenty-eight and fourteen candidate fusions are reported in SKBR3 and MCF7 samples, respectively, when the filtering parameters are changed to one spanning read and two supporting mate pairs. [file gb-2011-12-8-r72-S7.PDF]

SAMPLE ID	Fusion genes (left-right)	Chromosomes (left-right)	$5'$ position	$3'$ position	Spanning reads	Spanning pairs
SKBR3	TATDN1-GSDMB	8-17	125551264	38066175	311	555
MCF7	BCAS4-BCAS3	20-17	49411707	59445685	105	284
MCF7	ENSG00000254868-FOXA1	14-14	38184710	38061534	2	22
SKBR3	ANKHD1-PCDH1	5-5	139825557	141234002	4	15
SKBR3	SUMF1-LRRFIP2	3-3	4418012	37170638	3	12
SKBR3	CSE1L-ENSG00000236127	20-20	47688988	47956855	13	31
MCF7	RSBN1-AP4B1	1-1	114354329	114442495	6	7
MCF7	ARFGEF2-SULF2	20-20	47538545	46365686	17	20
MCF7	RPS6KB1-TMEM49	17-17	57992061	57917126	4	3
SKBR3	WDR67-ZNF704	8-8	124096577	81733851	3	3
MCF7	SULF2-ENSG00000171940	20-20	46415146	52210647	11	32
MCF7	ENSG00000224738-TMEM49	17-17	57184949	57915653	5	6
MCF7	ANKRD30BL-RPS23	2-5	133012791	81574161	2	6
MCF7	PAPOLA-AK7	14-14	96968936	96904171	3	3
SKBR3	ENSG00000251948-SLCO5A1	19-8	24184150	70602607	1	4
MCF7	SMARCA4-CARM1	19-19	11097267	11015625	1	3
MCF7	LRP1B-PLXDC1	2-17	142237963	37265642	2	5
SKBR3	PPEF2-ENSG00000248527	4-1	76807192	569235	1	3
SKBR3	CYTH1-EIF3H	17-8	76778283	117768257	18	37
SKBR3	FAM189A1-DCAF6	15-1	29525904	168007607	1	4
SKBR3	KLHDC2-SNTB1	14-8	50249310	121561198	2	4
SKBR3	ENSG00000252318-SLCO5A1	2-8	133038722	70602607	1	3
SKBR3	DIO2-ENSG00000249517	14-14	80669628	80854018	2	3
SKBR3	RARA-PKIA	17-8	38465535	79510590	1	5
SKBR3	RNF6-FOXO1	13-13	26795971	41192773	2	13
MCF7	PPM1D-USP32	17-17	58679978	58342772	2	4
SKBR3	BAT1-ENSG00000254406	6-11	31499072	119692419	2	30
SKBR3	PCBD2-ENSG00000252194	5-1	134260622	108113488	1	3
SKBR3	UNC45B-RPL8	17-8	33478265	146017770	1	3
SKBR3	FASN-PHKB	17-16	80041762	47539243	1	21
SKBR3	H19-MTRNR2L2	11-5	2017318	79946842	1	3
SKBR3	PCBD2-ANKRD30BL	5-2	134263179	133012790	1	5
MCF7	MTRNR2L8-PDE3A	11-12	10530124	20704373	1	3
SKBR3	S100A6-ENSG00000240409	1-1	153508431	569015	1	2
SKBR3	PCBD2-ENSG00000239776	5-12	134263289	127650986	2	3
SKBR3	ENSG00000225630-MTRNR2L8	1-11	565931	10530180	1	3
SKBR3	ENSG00000240409-PCBD2	1-5	569018	134260144	2	5
SKBR3	ENSG00000250815-OBFC2B	4-12	54794232	56619153	2	4
SKBR3	MTRNR2L2-PCBD2	5-5	79946305	134260456	2	2
SKBR3	ENSG00000205045-E2F3	17-6	33817342	20481454	2	3
MCF7	ENSG00000226505-MRPL36	2-5	70329650	1799907	5	20
SKBR3	CCDC85C-SETD3	14-14	100002351	99880270	5	6
