# Supplementary material for: TopHat-Fusion: an algorithm for discovery of novel fusion transcripts
Source: Genome Biol. 2011 Aug 11;12(8):R72. doi: 10.1186/gb-2011-12-8-r72 (PMC3245612; doi:10.1186/gb-2011-12-8-r72)
Supplement: Additional file 10 — Supplementary methods. [file gb-2011-12-8-r72-S10.DOC]

**Supplementary Methods**

In addition to finding fusion points using three (or more) segments as illustrated in Figure 3, TopHat-Fusion is able to identify fusions using two segments (the minimum number of segments required), and paired-end alignments are used to make this searching process more sensitive (additional file 11, Figure S2). By allowing a few mismatches when TopHat uses Bowtie to map segments from the initially unmapped (IUM) reads, it is possible that a segment will be mapped a few bases past a fusion point. This allows TopHat-Fusion to identify fusions with just two segments by realigning them to two chromosomes, or two different parts of a chromosome. Although this variation on the algorithm is less sensitive than the three (or more) segment approach, which allows middle segments to span a fusion point as well as a few boundary base pairs of the first and third segments, it turns out that this approach is quite effective considering the very deep coverage often available in RNA-seq data sets. As shown in additional file, Figure S2b, the alignment of a partner read is also used to identify a possible small range in which a fusion point may lie.

After identifying fusion points in the above step, and mapping segments against such fusions, it is necessary to connect the mapped segments to make a full read alignment, which is one of the most complicated processes in TopHat-Fusion. Given the mappings of the segments comprising a read, TopHat-Fusion stitches them together to produce full-length read alignments according to the following rules (illustrated in additional file 12, Figure S3). (1) Two consecutive segments of a read are aligned on the same chromosome with the same orientation, and the right genomic coordinate of a segment corresponds to the left coordinate of its subsequent segment or there is a junction or a deletion to fill the gap between two consecutive subsequences. (2) There is a fusion that connects the segments available. This stitching process is done by depth first search; i.e., given a first segment, TopHat-Fusion examines every second segment to check if any of them can be glued to the first one, and if there is such a second segment, it searches all the third segments. During the search process, an alignment of a segment may be reversed to have the same orientation with its preceding segment.
