# Supplementary material for: TopHat-Fusion: an algorithm for discovery of novel fusion transcripts
Source: Genome Biol. 2011 Aug 11;12(8):R72. doi: 10.1186/gb-2011-12-8-r72 (PMC3245612; doi:10.1186/gb-2011-12-8-r72)
Supplement: Additional file 12 — Figure S3 - stitching segments to produce a full read alignment. (a) The segment in the third row for segment 1 and the one in the first row for segment 2 are connected because they are on the same chromosome (i) in the forward direction and with adjacent coordinates. These are then matched to the second row in segment 3 and glued together, producing the full-length read alignment at the bottom. (b) TopHat-Fusion tries to connect the segment in the second row for segment 1 with segments in the first and second rows for segment 2, but neither succeeds. Case 1 would require two fusion points in the same read, and case 2 cannot be fused with consistent coordinates. (c) Attempts to connect the segment in the second row for segment 2 with the one in the first row in segment 3: in case 3, there is no intron available, there is no fusion in case 4, and case 5 would require more than one fusion. [file gb-2011-12-8-r72-S12.PDF]

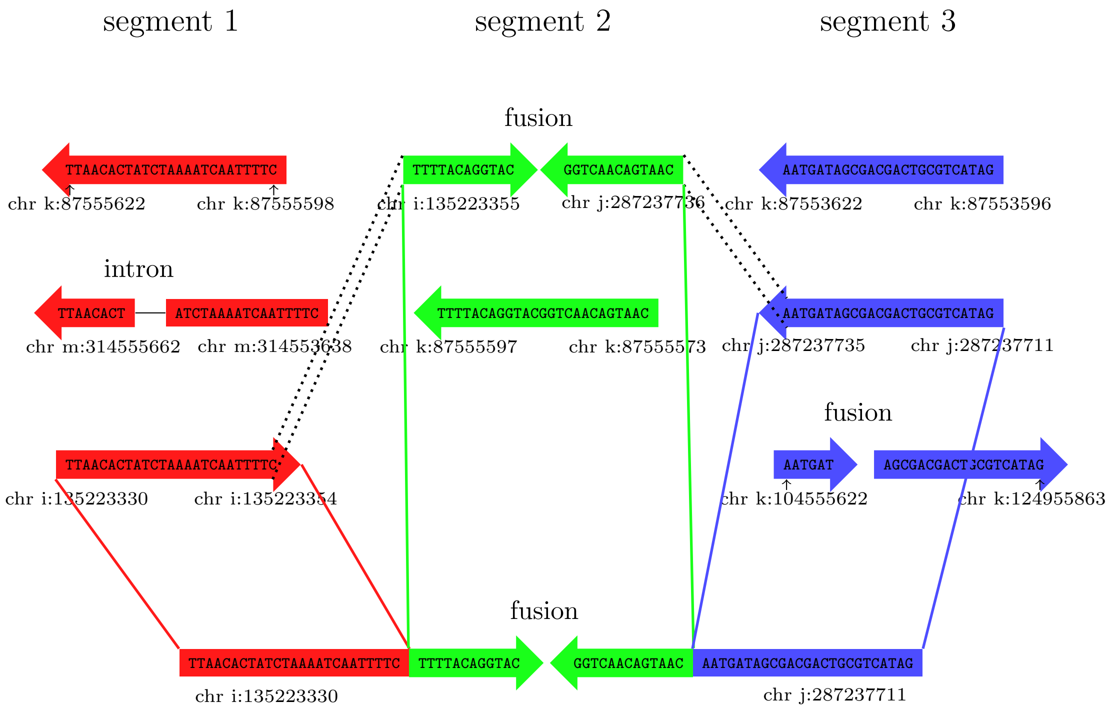

(a) a successful read alignment

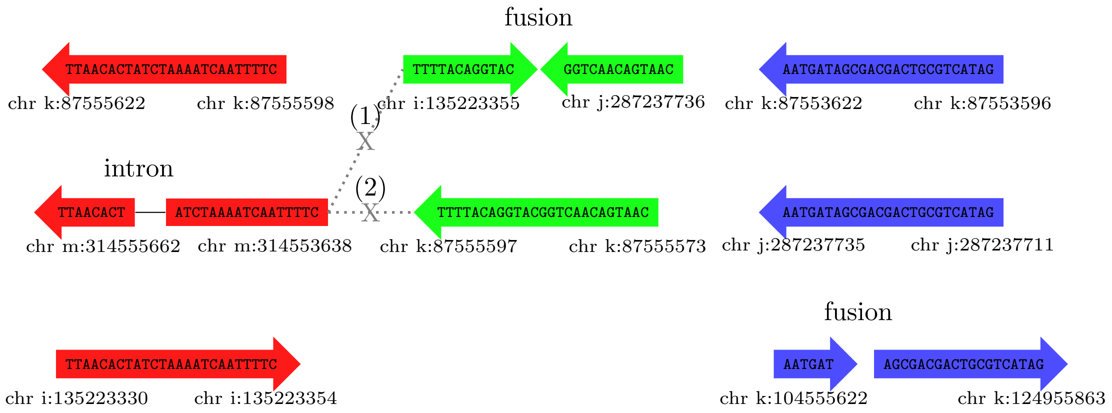

(b) A failure to connect first and second segments

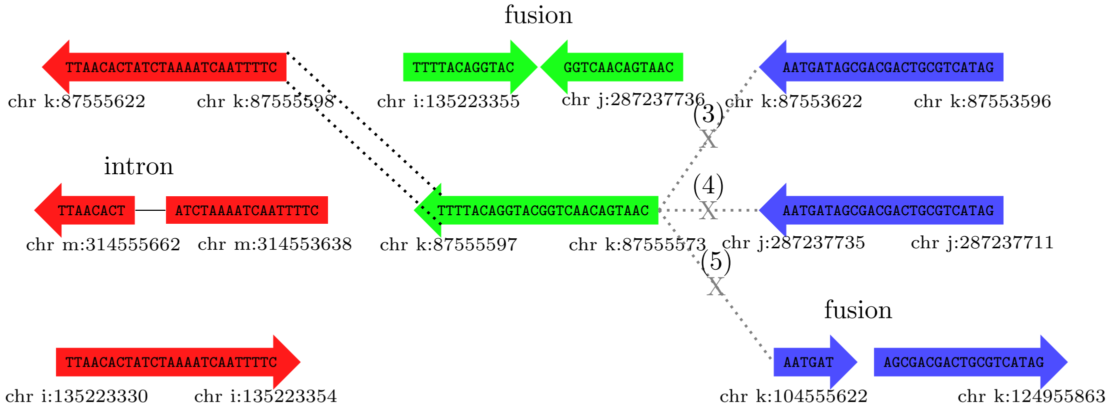

(c) A failure to connect second and third segments
